# Barriers to treatment for alcohol dependence: a qualitative study

**DOI:** 10.1186/1940-0640-8-S1-A5

**Published:** 2013-09-04

**Authors:** Sven Andréasson, Sara Wallhed Finn, Ann-Sofie Bakshi

**Affiliations:** 1 Karolinska Institute, Department of Public Health Sciences, Stockholm, Sweden

## 

Approximately half of all people with high alcohol consumption in Sweden fulfill criteria for either harmful consumption or dependence. Among the alcohol-dependent, the majority have dependence with low severity. They are also reluctant to seek treatment. This qualitative study, with data from seven focus group discussions and 14 individual interviews, aims to describe and explain how representations of alcohol consumption, dependence, and treatment create barriers to treatment. Thirty-two adults from the general population fulfilling DSM-IV criteria for alcohol dependence participated. Most of the participants agreed that they were heavy drinkers but did not perceive themselves as alcohol dependent. Having alcohol problems, as well as realizing the need for and entering treatment, was associated with shame and stigma, producing a strong barrier to treatment. The participants’ knowledge about treatment was limited and somewhat faulty, as they thought that treatment mainly involves medication with disulfiram, lifelong abstinence, and inpatient care at rehabilitation clinics. As these treatments are socially restrictive and stigmatizing, this understanding created a barrier to treatment. While treatment for alcohol problems in primary care was seen as less stigmatizing, the expertise among general practitioners in this field was questioned. Results indicate that, to lower the threshold for treatment seeking, treatment services need to better match the needs and wishes of the potential service users as well as take stigmatization into account. Primary care practices and general practitioners need to market their ability to treat people with drinking problems. The clinical understanding of alcohol dependence needs to be expanded to include mild to moderate dependence, conditions which can be managed in primary care.

